# The Optimal Management of Patients with Atrial Fibrillation and Acute Heart Failure in the Emergency Department

**DOI:** 10.3390/medicina59122113

**Published:** 2023-12-02

**Authors:** Maria Velliou, Elias Sanidas, Antonis Diakantonis, Ioannis Ventoulis, John Parissis, Effie Polyzogopoulou

**Affiliations:** 1 Emergency Medicine Department, Attikon University Hospital, 12462 Athens, Greece; maravelliou84@yahoo.gr (M.V.); antoniosdiak@yahoo.gr (A.D.); jparissis@yahoo.com (J.P.); 2 Department of Cardiology, Laiko General Hospital, 11527 Athens, Greece; easanidas@yahoo.gr; 3 Department of Occupational Therapy, University of Western Macedonia, 50200 Ptolemaida, Greece; iventoulis@uowm.gr

**Keywords:** atrial fibrillation, acute heart failure, cardiogenic shock, emergency department, acute rate control, rhythm control, cardioversion

## Abstract

Atrial fibrillation (AF) and acute heart failure (AHF) are two closely interrelated conditions that frequently coexist in a manifold manner, with AF serving either as the causative factor or as the consequence or even as an innocent bystander. The interplay between these two clinical conditions is complex, given that they share common pathophysiological pathways and they can reciprocally exacerbate each other, thus triggering a vicious cycle that worsens the prognosis and increases the thromboembolic risk. The optimal management of AF in the context of AHF in the emergency department remains a challenge depending on the time onset, as well as the nature and the severity of the associated symptoms. Acute rate control, along with early rhythm control, when indicated, and anticoagulation represent the main pillars of the therapeutic intervention. The purpose of this review is to elucidate the pathophysiological link between AF and AHF and accordingly present a stepwise algorithmic approach for the management of AF in AHF patients in the emergency setting.

## 1. Introduction

Atrial fibrillation (AF) is the most common cardiac arrhythmia in patients with chronic heart failure (HF), increasing four to six times the risk of hospitalization for acute HF (AHF). The relationship between these two clinical conditions is complex, as AF can exacerbate left ventricular (LV) dysfunction and vice versa; either an episode of AF itself can precipitate the worsening of HF in a previous stable patient or the occurrence of AHF per se can generate an episode of AF with rapid ventricular response [1,2]. However, it is not always easy to differentiate whether AF is the primary cause or the consequence of AHF. The MultiSENSE (Multisensor Chronic Evaluation in Ambulatory HF Patients) study showed that newer implantable cardiac devices in HF patients could shed light on the detection of causation by monitoring several physiological sensors capable of quantifying HF status. Hence, treating HF deterioration could probably prevent AF episodes [3].

Accumulative data from large-scale studies and registries indicate that AF is present in approximately 35% of the patients who are admitted to the emergency department (ED) with signs and/or symptoms suggestive of HF deterioration (Table 1) [4,5,6,7,8,9,10,11,12,13], while 20% experience a new episode of AF during hospitalization [14,15,16]. In the context of AHF, AF represents an independent predictor of morbidity, all-cause mortality and adverse in-hospital outcomes. Patients with both AF and AHF usually show less improvement in dyspnea during the first hours of in-hospital treatment and they have a more protracted course of in-hospital stay as well as higher rates of HF rehospitalization. In addition, these patients are usually discharged to a facility rather than their home [13].

The ED is the first access point in the healthcare system where the optimal management of this subgroup of patients can be achieved in order to improve the prognosis both in the short- and long-term [17,18]. The aim of this review is to elucidate the intricate pathophysiological link between AF and AHF and highlight the basic principles of the initial treatment strategy of AF across the diverse spectrum of AHF in the emergency setting.

## 2. The Interplay between AF and AHF

Three main scenarios exist to describe the complex interplay between AF and AHF: (1) AF as the triggering factor of AHF, (2) AF as the consequence of AHF and (3) AF as an innocent bystander of AHF [19].

### 2.1. AF as the Triggering Factor of AHF

The occurrence of AF can cause substantial clinical deterioration of a patient with already existing HF, resulting in the so-called acute decompensated HF and subsequently provoking the transition to advanced HF. Furthermore, when longstanding, AF with a high ventricular rate can lead to the development of de novo HF in a previously ‘‘HF-naïve’’ patient, a condition often termed tachycardia-mediated cardiomyopathy. In either case, regardless of the clinical scenario, AF is considered one of the potential etiological and precipitating factors of AHF (represented by the letter “A” in the acronym CHAMPIT, standing for arrhythmia) that should be identified and treated at the very early stage [2].

From a pathophysiological point of view, AF induces ventricular remodeling, both cellular and extracellular, which is predominantly characterized by altered collagen distribution, increased collagen density and thickening of collagen fibers. All these structural abnormalities result in the dilation and thinning of the ventricular walls, accompanied by increased LV mass and myocardial stiffness, which collectively lead to decreased cardiac contractility and impaired diastolic relaxation, hence potentiating the development of HF [20]. Moreover, the atria fail to eject blood into the ventricles effectively due to the increased and irregular heart rate, the resultant atrioventricular (AV) asynchrony and the loss of the atrial kick. The atrial kick corresponds to the last phase of the ventricular diastolic period and contributes to the remaining 20–30% of the total ventricular end-diastolic volume. The loss of the atrial kick results in limited ventricular filling, increased ventricular filling pressures, reduced cardiac contractility, compromised stroke volume, functional mitral regurgitation and diastolic dysfunction. Cardiac output is usually decreased by 20–30% or more, while systemic vascular resistance is increased [21,22]. In addition, an increased heart rate elevates ventricular end-diastolic pressure and decreases diastolic filling time, thus further diminishing cardiac performance. This chain of events culminates in the deterioration of the patient’s clinical status by disrupting the vulnerable HF equilibrium and leading to varying levels of HF acuity [23]. Furthermore, the rapid ventricular rate in patients with AF impairs myocardial perfusion by decreasing diastolic coronary blood flow, eventually causing or worsening myocardial ischemia [24]. Moreover, AF is associated with hyperactivity of the sympathetic nervous system (SNS), which is per se considered another fundamental mechanism that triggers the progression of HF [25,26]. Last but not least, AF is correlated with increased aortic stiffness, which in turn leads to increased LV afterload and elevated peripheral vascular resistance, both of which further compromise cardiac performance and pose an additional risk of HF exacerbation [27].

### 2.2. AF as the Consequence of AHF

**Figure 1:**
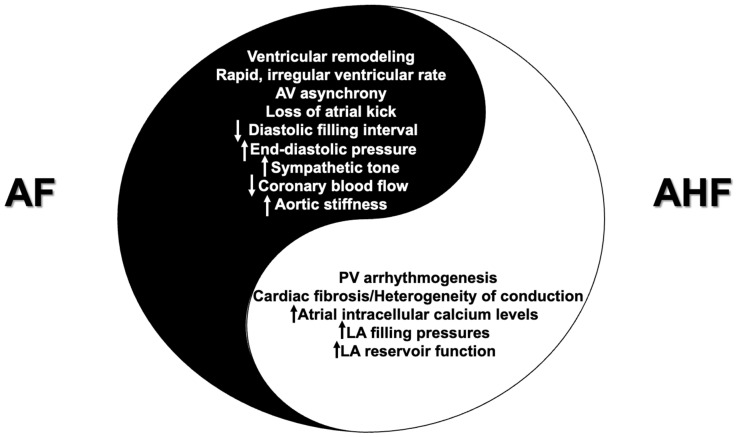
The vicious pathophysiological cycle between AF and AHF. Abbreviations: AHF: acute heart failure; AF: atrial fibrillation, AV: atrioventricular; LA: left atrial; PV: pulmonary vein.

HF creates a proarrhythmic environment that favors the generation and maintenance of AF. An experimental study showed that the enhanced arrhythmogenesis in the pulmonary vein (PV) region might be a potential mechanism of HF-induced AF. Indeed, PV cardiomyocytes in rabbits with experimentally induced HF were characterized by a higher incidence of delayed afterdepolarization and faster spontaneous activity, compared to controls without HF [28]. Additionally, HF results in defective calcium (Ca2+) handling, owing to the dysregulation of the ryanodine receptor in cardiac myocytes and the dysfunction of the sarcoplasmic reticulum Ca2+-ATPase. The impaired intracellular Ca2+ cycling, with increased Ca2+ diastolic leak and decreased Ca2+ reuptake from the sarcoplasmic reticulum, translates into increased intracellular calcium levels, which can trigger aberrant depolarizations and arrhythmias, thus further contributing to the development and progression of AF [29,30]. Furthermore, HF is associated with a proinflammatory milieu which, in conjunction with the activation of the SNS and the renin-angiotensin-aldosterone system (RAAS), results in the development of cardiac fibrosis, marked by heterogeneity in atrial conduction and regions with slow electrical conduction [20]. Additionally, increased LV filling pressures due to HF are transmitted to the left atrium (LA) causing elevated intra-atrial pressures, increased LA stiffness and reduced LA reservoir function [31,32]. Figure 1 illustrates the vicious pathophysiological cycle between AF and AHF.

### 2.3. AF as an Innocent Bystander of AHF

In the event of AF with moderate ventricular response, AF could be considered as an innocent bystander of AHF. In this scenario, the ventricular rate of AF ranges between 60 and 100 beats per minute (bpm) and rarely causes hemodynamic instability. Thus, the treatment of AHF is more critical than the treatment of the arrhythmia and the emergency physician should seek for another precipitant, other than AF, for the worsening of HF [33].

## 3. The Management of AF in AHF Patients in the ED

The management of AF per se in the context of AHF in the ED is complicated depending on the time onset of the arrhythmia, as well as the nature and the severity of the associated symptoms. In any case, the three main therapeutic pillars include acute rate control, early rhythm control, when indicated, and anticoagulation [1,2].

### 3.1. Rate Control

High heart rate on admission is a strong independent predictor of adverse outcomes and all-cause mortality in patients with AHF [34]. Therefore, acute rate control remains a crucial part in the management of patients with AHF and concomitant AF, in an effort to decrease ventricular rate, improve ventricular filling and maintain adequate cardiac output [35]. The optimal target during rate control is not clear and should be individualized and guided by the hemodynamic and clinical improvement. Probably, rates lower that 100 bpm might not be realistic during the initial management of an AHF episode, at least not until hypoxia and volume overload have been restored; nevertheless, a ventricular rate below 100–110 bpm is usually recommended [21].

In hemodynamically unstable patients, urgent electrical cardioversion (ECV) should be performed. In stable patients, the choice of drug for rate control is usually dictated by the patient’s characteristics and comorbidities, clinical symptoms, LV function and hemodynamics [19].

The role of beta-blockers in AHF is controversial due to their negative inotropic effect [36]. The 2021 HF guidelines by the European Society of Cardiology (ESC) recommend that beta-blockers should be cautiously initiated in hospitalized patients after hemodynamic stabilization, relief of congestion and restoration of euvolemia have been achieved (but, ideally, they should be initiated before discharge) [2]. Nonetheless, the rapid-acting, highly cardioselective β1-receptor blocker, landiolol, has emerged as a promising option for rapid rate control in AHF patients with coexisting AF. Landiolol exhibits weak negative inotropic effects and fast pharmacokinetic properties, having only a minor impact on blood pressure. All these characteristics render the drug more suitable for the therapeutic management of critically ill patients with comorbidities [37].

The landmark trial that aimed to evaluate the efficacy and safety of landiolol in patients with AHF and atrial tachyarrhythmias, including AF and atrial flutter, was the prospective, multicenter J-Land (Japanese landiolol versus digoxin) study, which was conducted in 95 hospitals in Japan. The J-Land study showed that continuous intravenous administration of landiolol was superior to digoxin for urgent rate control among patients with AF or atrial flutter with rapid ventricular response (heart rate ≥ 120 bpm), LV ejection fraction (LVEF) 25–50% and New York Heart Association (NYHA) class III or IV. Successful rate control (≥20% decrease in heart rate from baseline and less than 110 bpm at two hours after the therapeutic intervention) was achieved in 48% of those who received landiolol and in only 13.9% of those who received digoxin. Concerning the safety of the drugs, there was no statistically significant difference between the two studied groups [38]. A subgroup analysis of the J-Land study also revealed that the beta-blocker was superior to digoxin irrespective of the patients’ baseline characteristics such as age, gender, heart rate, systolic blood pressure, LVEF, previous intake of oral beta-blockers and renal function [39]. Thereafter, numerous other trials confirmed the aforementioned findings, highlighting the beneficial role of landiolol in patients with rapid AF and AHF [40,41,42,43,44,45].

Esmolol, a selective β1-receptor blocker, represents an alternative for the acute rate control of AF in the emergency setting since it is rapid-acting, easily titrated, inexpensive and has a good tolerability profile. Nevertheless, esmolol demonstrates a greater negative inotropic effect and produces a greater reduction in blood pressure compared to landiolol. In addition, the more delayed onset of action and the less pronounced heart rate-lowering effect of esmolol in comparison to landiolol may limit the administration of esmolol in the context of AHF [46].

The non-dihydropyridine calcium channel blockers (CCBs), namely, verapamil and diltiazem, are recommended for rate control in patients with LVEF > 40% [1]. These drugs link to the L-type calcium channels of the cardiac myocytes, inhibiting the inflow of Ca2+ ions and, hence, slowing the conduction through the AV node, thereby resulting in the control of the ventricular rate. Both verapamil and diltiazem have low cost and minimum adverse events, while their peak therapeutic effect occurs within five minutes after their intravenous administration [47].

A retrospective, single-center study of 73 patients with AF and rapid ventricular response (heart rate at baseline: 139.0 ± 18.3 bpm) compared the effectiveness of diltiazem, verapamil and metoprolol in terms of achieving a ventricular rate less than 100 bpm and showed that there was no statistically significant difference between the three agents after one hour of intravenous administration. The median time to achieving acute rate control was 166 min for those who received diltiazem, 100.5 min for those who received verapamil and 297 min for those who received metoprolol. Compared to non-dihydropyridine CCBs, the metoprolol group experienced hypotension more frequently, had higher rates of admission to the intensive care unit (ICU) and required the addition of a second rate-controlling agent more often [48].

Available evidence indicates that both intravenous diltiazem and verapamil are equally effective in achieving heart rate control in patients with rapid AF. A retrospective, case–control study which included 146 patients, 5% of whom had comorbid HF, showed that 90% of the patients in the diltiazem group and 89% of those in the verapamil group accomplished ventricular rates less than 110 bpm. Only 4% in the diltiazem group and 1.4% in the verapamil group required the addition of amiodarone infusion on top of non-dihydropyridine CCBs in order to achieve a heart rate less than 100 bpm. There was no significant difference between the two treatment groups regarding the need for inotropes/vasopressors and the incidence of hypotension (defined as systolic blood pressure < 90 mmHg) or bradycardia [49]. Likewise, a study of 182 patients with AF or atrial flutter, 10% of whom had LVEF < 50%, demonstrated that the safety and efficacy outcomes were similar for verapamil and diltiazem when used for acute rate control [50].

Amiodarone is a safe and effective option for acute rate control in patients with AF and AHF with severely depressed LVEF or in hemodynamically unstable patients [1]. This class III antiarrhythmic drug exerts minor negative inotropic effects. Short-term intravenous administration slows conduction velocity through the AV node and prolongs the refractory period of the AV node, while it has a minor impact on the refractoriness of atrial and ventricular myocytes as well as the His–Purkinje system, thus causing no significant overall increase in QRS duration and QT interval. Furthermore, acute venodilation following intravenous use results in preload reduction. The most important side effects of amiodarone are hypotension, occurring in up to 30% of patients, and thrombophlebitis. Thus, administration of amiodarone via a central venous line and appropriate drug dilution are warranted [51].

A study of 100 patients with rapid AF (>135 bpm at baseline), among whom 12% had a history of HF, showed that intravenous amiodarone was more effective than intravenous digoxin in controlling acute heart rate. After an hour, the heart rate was 94 ± 22 bpm in the amiodarone group compared to 105 ± 22 bpm in the digoxin group [52]. In line with the aforementioned findings, a small study of 20 consecutive patients with AHF and AF (mean heart rate at baseline: 137 ± 15 bpm) revealed that intravenous amiodarone effectively controlled the ventricular rate irrespective of the type of AF (recent onset, paroxysmal, persistent or long-standing AF). Moreover, AF was converted to sinus rhythm in 91% of patients with recent onset or paroxysmal AF after rate control and within 5.8 h. Of note, AF was not converted in any patient with persistent or long-standing AF, though amiodarone reduced the heart rate to a mean of 104 bpm. No serious side effects, such as bradycardia or marked hypotension, were recorded [53].

Digoxin is a well-established, cheap and widely available drug that exerts positive inotropic effects. It also possesses parasympathetic properties, thereby slowing the electrical conduction through the AV node and lowering the heart rate [54]. Current guidelines recommend it for rate control in patients with AF and especially those with concomitant HF [1,2]. A single-center, retrospective cohort study of 210 patients with AHF triggered by tachyarrhythmia (AF/atrial flutter) showed that digoxin, given intravenously at a dose of 0.75 mg or more, was very effective in achieving rate control. Indeed, the heart rate substantially decreased from 141 ± 22 bpm at baseline to 101 ± 23 bpm within 24 h and to 98 ± 22 bpm within 48 h. Only one patient experienced nausea, while no statistically significant difference was observed between predicted and observed 30-day mortality [55]. However, digoxin’s delayed onset of action (approximately 3–6 h), owing to its prolonged distribution phase, limits the use of this agent in the emergency setting [56]. Therefore, given the availability of other rate-controlling drugs in the ED, digoxin might not be an optimal choice as monotherapy, but it can be used as an effective adjunctive agent on top of other therapies [19].

### 3.2. Rhythm Control

Successful restoration and maintenance of sinus rhythm in patients with AF is associated with the prevention of irreversible atrial remodeling and a lower risk of cardiovascular events and mortality [57]. The prospective multicenter KorAHF (Korean AHF) registry, which included 1961 patients presenting to the ED with AF and signs and/or symptoms suggestive of AHF, demonstrated lower rates of HF readmission and all-cause mortality in the group of patients in whom sinus rhythm was restored compared to the group of patients with persistent AF [58].

Early cardioversion is indicated in patients with recent onset AF, ideally when AF duration is less than 48 h, after consideration of the thromboembolic risk. In patients who are not on chronic oral anticoagulation therapy or in patients with AF onset >48 h, a transesophageal echocardiography before cardioversion or at least three weeks of therapeutic anticoagulation are warranted. Rhythm control is contraindicated in patients with permanent AF [1,2].

In hemodynamically unstable patients, synchronized ECV is recommended for acute rhythm control and immediate restoration of sinus rhythm. In hemodynamically stable patients, either ECV or pharmacological cardioversion can be performed. ECV is correlated with high success rates in terms of immediate rhythm restoration (>90% of new onset or paroxysmal AF cases) and reduced length of hospital stay. On the other hand, pharmacological cardioversion does not require sedation and analgesia. The selection of the mode of elective cardioversion is influenced by local practices and hospital protocols and is subject to geographic variations [1,2,59]. The RHYTHM-AF trial revealed that Sweden, Australia, United Kingdom and Germany adopted ECV as the main strategy, whereas Italy, Spain and Brazil had a strong preference for pharmacological cardioversion [60]. Moreover, a survey, which was conducted by the European Heart Rhythm Association (EHRA) and analyzed data from 57 centers across Europe, showed that ECV was the preferred mode of elective cardioversion in 67.9% of the overall sites; only 18.8% of the centers adopted pharmacological cardioversion as their principal approach [61].

The choice of the agent used for pharmacological cardioversion is determined by the presence and the severity of the patient’s underlying heart disease. Amiodarone is the best antiarrhythmic drug in patients with significant structural heart disease, particularly those suffering from HF with LV systolic dysfunction. This is due to the fact that amiodarone has a favorable safety profile and carries a low risk of proarrhythmia. The only disadvantage is its delayed onset of action (8–24 h) [1,2,59].

Vernakalant has emerged as an alternative means of pharmacological conversion. Its action is predominantly exerted on atrial myocytes, since it possesses high affinity for ion channels involved in the repolarization process of atrial tissue. Owing to its electrophysiological selectivity for atrial cells, vernakalant exerts minimum effects on the ventricular tissue and thus carries a relatively low risk of ventricular proarrhythmia [62]. Vernakalant is considered the most effective antiarrhythmic drug in terms of achieving rapid cardioversion of AF. In fact, the vast majority of patients (75–82%) convert to sinus rhythm after the first dose of vernakalant within a median time of 8–14 min [63]. According to available data, vernakalant is superior to amiodarone for the acute conversion of recent onset AF and it has been associated with a higher rate of symptom relief [64]. It can be used in patients with mild HF, whereas it is contraindicated in those with an acute coronary syndrome, NYHA III or IV HF, prolonged QT or severe aortic stenosis [1].

The SPECTRUM (Surveillance of Pharmacologic thErapy for Cardioversion in aTrial fibrillation Registry Using IV treatMent) study assessed the effectiveness and safety of intravenous vernakalant in a large cohort of 1778 patients with recent onset AF (<7 days). Approximately 3% of the recruited patients had a history of HF and the presenting symptoms were palpitations, dyspnea, dizziness, light-headedness, chest pain, syncope and near syncope. In 70% of the study population, conversion to sinus rhythm with vernakalant was achieved within 12 min from the onset of infusion and within 11 h from the onset of AF. Symptomatic bradycardia was the most common side effect. Neither deaths nor cases of sustained ventricular tachycardia, ventricular fibrillation or torsade de pointes were recorded [65].

Last but not least, antazoline, a first-generation antihistaminic agent with quinidine-like antiarrhythmic properties, seems to be a promising drug for the pharmacological cardioversion of recent onset AF [66]. The exact mechanism of action is not fully elucidated yet. Experimental data has shown that antazoline increases atrial action potential duration and effective refractory period, atrial and ventricular postrepolarization refractoriness and interatrial conduction time. Blocking both potassium and sodium ion channels might also be responsible for the antiarrhythmic effects [67,68]. The CANT (Cardioversion with Intravenous Antazoline Mesylate) II study, a multicenter, retrospective real-world registry, demonstrated that antazoline is noninferior to other available antiarrhythmic drugs, including amiodarone and propafenone, in terms of successful rhythm conversion among 1365 patients admitted to the ED with new onset AF, 9.4% of whom had an LVEF < 50%. Concerning the safety profile, the rate of serious adverse events, such as bradycardia below 45 bpm, hypotension, syncope or death, was higher in the antazoline group compared to the amiodarone group (5.2% versus 2.1%), but it was comparable between the antazoline group and the propafenone group (5.2% versus 7.3%) [69]. However, antazoline is not formally listed in the latest published ESC guidelines for the management of AF [1]. The current evidence is lacking and further research is warranted in this field.

Regarding other antiarrhythmic drugs, such as ibutilide, propafenone and flecainide, they are not recommended for patients with HF [1,2].

### 3.3. Anticoagulation

HF confers additional thrombotic risk in patients with AF. On the grounds of this, HF is included in the risk score CHA_2_DS_2_VASc. A large meta-analysis of 71 studies showed that the relative risk of in-hospital venous thromboembolism among patients hospitalized due to HF was 1.5, while the use of anticoagulants reduced the risk [70]. Both AF and HF guidelines by the ESC suggest the use of low-molecular-weight heparin, unfractionated heparin or oral anticoagulants in all patients who present to the ED with AHF and concomitant AF, unless there are contraindications or the patient is already on chronic anticoagulant therapy [1,2]. Anticoagulation also minimizes the risk of acute thromboembolism after cardioversion in patients who are not on chronic treatment [71].

**Figure 2:**
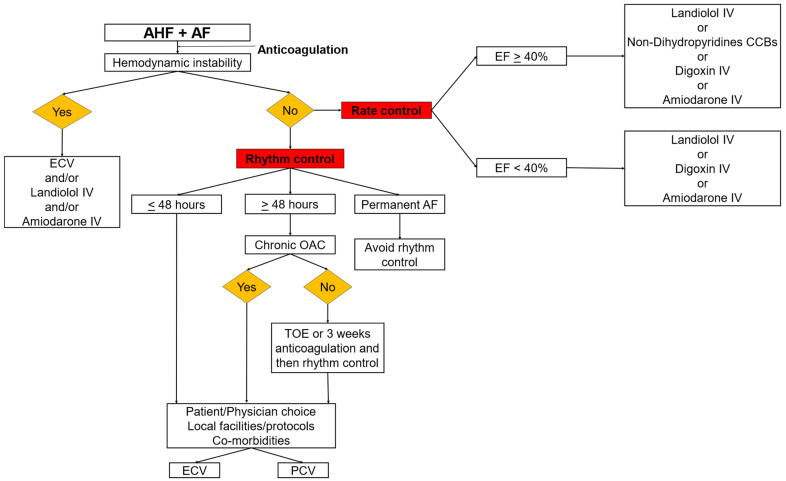
An algorithmic approach for the management of AF in the context of AHF in the emergency department. Abbreviations: AF: atrial fibrillation; AHF: acute heart failure; CCBs: calcium channel blockers; ECV: electrical cardioversion; EF: ejection fraction; IV: intravenous; OAC: oral anticoagulation; PCV: pharmacological cardioversion; TOE: transesophageal echocardiography.

Figure 2 displays a proposed stepwise algorithmic approach for the optimal management of AF in the context of AHF in the emergency setting.

## 4. Early Rhythm Control versus Acute Rate Control

Over the course of the last two decades, there has been a debate whether a patient with AF is better served with a rate or rhythm control strategy. Most trials have demonstrated that there is no difference between the two treatment strategies with regard to the primary endpoints, including all-cause and cardiovascular mortality, as well as the secondary endpoints, such as the quality of life [72,73,74,75,76,77,78].

Recently, however, several studies have highlighted the benefits of early initiation of rhythm control therapy and prompt restoration of sinus rhythm in patients with new onset or paroxysmal AF. This treatment strategy aims at the prevention of irreversible structural changes in the atria, while reducing the risk of adverse cardiovascular outcomes, such as stroke and mortality [79,80,81,82].

The EAST-AFNET 4 trial (Early Treatment of Atrial Fibrillation for Stroke Prevention Trial) was a multicenter randomized study from 135 sites in 11 European countries. It included 2789 patients with early AF (median time since diagnosis: 36 days) and underlying cardiovascular conditions, such as HF (almost 29% of the study population), a previous transient ischemic attack or stroke, arterial hypertension, diabetes mellitus, severe coronary artery disease, chronic kidney disease (estimated Glomerular Filtration Rate, eGFR: 15–59 mL/min/1.73 m^2^) and/or LV hypertrophy. This study showed that the early rhythm control strategy was correlated with a lower risk of adverse cardiovascular events compared to the usual care, which entailed rate control without rhythm control therapy. The primary endpoint, defined as a composite of cardiovascular death, stroke or hospitalization due to deterioration of HF or acute coronary syndrome, was met in 249 patients in the early rhythm control group, as opposed to 316 patients in the usual care group [79]. Of note, according to a subanalysis of the EAST-AFNET 4 trial, the clinical benefit of early rhythm control therapy was also observed in patients with new onset AF and concomitant HF, irrespective of the HF subtype [80].

A post hoc analysis of the ATHENA trial (a placebo-controlled, double-blind, parallel arm trial to assess the efficacy of dronedarone 400 mg bid for the prevention of cardiovascular hospitalization or death from any cause in patients with atrial fibrillation/atrial flutter) demonstrated that early rhythm control with dronedarone resulted in significantly fewer cardiovascular events compared to placebo in patients with new onset AF, after applying the EAST-AFNET 4 inclusion and exclusion criteria [81]. Likewise, an analysis of the UK Biobank (UKB) database, which included propensity-score matching of patients with AF (52% with a history of HF) who were considered eligible based on the EAST-AFNET 4 criteria, revealed that the early rhythm control strategy was safe in routine care and should be considered in the management of patients with recent onset AF [82].

Therefore, early rhythm control might be considered the first step in the management of patients with AHF and new onset AF, whenever AF is the consequence of HF deterioration. On the other hand, acute rate control might be the preferred treatment strategy in patients with AHF and a history of AF, in whom the arrhythmia is the precipitant of HF worsening.

## 5. AF in Cardiogenic Shock

AF results in further deterioration of the hemodynamic status of patients with cardiogenic shock (CS), owing to the lack of atrial systole and the impaired diastolic filling, in conjunction with the elevated LA pressures and the activation of the neurohormonal system [32]. Urgent ECV under conscious sedation and analgesia, coupled with anticoagulation, should be the first step in the therapeutic management of patients in CS complicated with rapid AF. Amiodarone could be administered for cardioversion to sinus rhythm, if clinically indicated, or for acute rate control. The major disadvantage of intravenous amiodarone is that it further decreases blood pressure, thereby worsening the already compromised hemodynamic profile [1]. Landiolol has emerged as an alternative drug that can be used for acute rate control in patients with CS. Cumulative data derived from retrospective studies [83,84,85,86,87] and case reports [88,89,90] supports the use of landiolol in the acute management of patients with CS and rapid AF, given that it is considered to be an effective and safe adjunctive agent that does not intervene with the concurrent administration of inotropes and vasopressors. Low-dose landiolol significantly reduces the heart rate by 11–22%, decreases pulmonary capillary wedge pressure (PCWP) and increases mixed venous oxygen saturation (SvO2). Moreover, it increases stroke volume and improves cardiac contractility without affecting blood pressure, mean right atrial pressures, systemic vascular resistance or cardiac index. Notably, landiolol also seems to be helpful in the conversion of AF to sinus rhythm [37].

## 6. Conclusions

AF occurs in approximately one third of patients with AHF and is associated with unfavorable prognosis and increased morbidity and mortality. The optimal management of patients presenting to the ED with AF and AHF requires an integrated approach. Acute rate control remains the main therapeutic intervention for all patients with rapid ventricular response, especially for those with a history of AF, in whom the arrhythmia is the causative factor of HF deterioration. A ventricular rate below 100–110 bpm should be the ideal target. Early rhythm control by means of electrical or pharmaceutical cardioversion is recommended in patients with new onset AF, including those in whom the arrhythmia is the consequence of HF deterioration. In hemodynamically unstable patients, urgent ECV should be performed. Last but not least, stroke prevention with the administration of low-molecular-weight heparin, unfractionated heparin or oral anticoagulants is another important mainstay in the treatment of patients with AHF and AF. In order to achieve the optimal management of patients with AHF and AF in the emergency setting, it is crucial for the emergency physician to abide by the aforementioned main therapeutic pillars, which should be pursued and addressed in a consistent and timely manner.

## Figures and Tables

**Table 1:** The prevalence of AF in the setting of AHF from different large-scale registries.

Study	Patients	Age (years)	History of HF (%)	AF (%)
EHFS I, 2003 [4]	11,327	71.0	65	43
ADHERE, 2003 [5]	105,388	72.0 ± 14.0	75	31
EHFS II, 2006 [6]	3580	69.9 ± 12.5	63	38.7
OPTIMIZE-HF, 2007 [7]	48,612	73.1 ± 14.2	87	31
ESC-HF Pilot (AHF arm), 2010 [8]	1892	70.0 ± 13.0	75	43.7
ALARM-HF, 2011 [9]	4953	66.0–70.0	64	24.4
THESUS-HF, 2012 [10]	1006	52.3 ± 18.3	NA	18.3
ATTEND, 2013 [11]	4842	>20	36.2	39.6
THER, 2015 [12]	1232	61.2 ± 13.6	100	14.7
ASCEND, 2016 [13]	7007	56.0–76.0	NA	De novo AF 38.2 History of AF 61.8

Abbreviations: AF: atrial fibrillation; AHF: acute heart failure; HF: heart failure; NA: not available. EHFS: EuroHeart Failure Survey; ADHERE: Acute Decompensated Heart Failure National Registry; OPTIMIZE-HF: Organized Program to Initiate Lifesaving Treatment in Hospitalized Patients With Heart Failure; ESC-HF Pilot: European Society of Cardiology Heart Failure Pilot Registry; ALARM-HF: Acute Heart Failure Global Registry of Standard Treatment; THESUS-HF: Sub-Saharan Africa Survey of Heart Failure; ATTEND: Acute Decompensated; Heart Failure Syndromes; THER: Trivandrum Heart Failure Registry; ASCEND: Acute Study of Clinical Effectiveness of Nesiritide in Decompensated Heart Failure.

## Data Availability

No new data were created or analyzed in this study.
